# Fuzzy Optimization on the Synthesis of Chitosan-Graft-Polyacrylic Acid with Montmorillonite as Filler Material: A Case Study

**DOI:** 10.3390/polym11040738

**Published:** 2019-04-23

**Authors:** Angelo Earvin Sy Choi, Cybelle Morales Futalan, Jurng-Jae Yee

**Affiliations:** 1 National Research Center for Disaster-Free and Safe Ocean City, Busan 49315, Republic of Korea; angelochoi2003@yahoo.com (A.E.S.C.); cmfutalan@gmail.com (C.M.F.); 2 Department of Architectural Engineering, Dong-A University, Busan 49315, Korea

**Keywords:** ammonium persulfate, fuzzy optimization, *N*,*N*′-methylenebisacrylamide, Pareto set, polyacrylic acid, swelling capacity, variable cost

## Abstract

In this paper, the synthesis of a chitosan–montmorillonite nanocomposite material grafted with acrylic acid is presented based on its function in a case study analysis. Fuzzy optimization is used for a multi-criteria decision analysis to determine the best desirable swelling capacity (*Y_Q_*) of the material synthesis at its lowest possible variable cost. For *Y_Q_*, the integrating the result’s cumulative uncertainty is an essential element to investigate the feasibility of the developed model equation. The Pareto set analysis is able to set the appropriate boundary limits for *Y_Q_* and the variable cost. Two case studies are presented in determining the lowest possible cost: Case 1 for maximum *Y_Q_*, and Case 2 for minimum *Y_Q_*. These boundary limits were used in the fuzzy optimization to determine its global optimum results that achieved the overall satisfaction ratings of 67.2% (Case 1) and 52.3% (Case 2). The synthesis of the polyacrylic acid/chitosan material for Case 1 resulted in 305 g/g *Y_Q_* and 10.8 USD/kg, while Case 2 resulted in 97 g/g *Y_Q_* and 12.3 USD/kg. Thus, the fuzzy optimization approach proves to be a practical method for examining the best possible compromise solution based on the desired function to adequately synthesize a material.

## 1. Introduction

Superabsorbents are smart, functional materials composed of lightly crosslinked hydrophilic polymers with the capacity to hold a large quantity of aqueous and biological fluids even at extreme pressure [1]. It also has the capability to release the collected fluids under a dry environment [2]. Superabsorbents are characterized by their high swelling capacities where saline fluids and water are retained up to 100–1000 times their original dry weight without losing their structural framework and integrity [3]. The preparative methods for superabsorbents involve the attachment of various functional groups such as amide, amine, hydroxyl, sulfonic acid, and carboxylic acid onto the polymer network [4]. Excellent properties of superabsorbents include elasticity, hydrophilicity, high swelling rate, porosity, softness and biodegradability [5,6,7]. Superabsorbents are classified into three groups: synthetic (derived from petroleum products), natural, and hybrid (vinyl monomers grafted onto natural polymers) [8]. However, petroleum-based polymers have limited utilization due to poor degradability, costly to produce, and toxicity with detrimental impacts to human health and the environment [9,10]. 

Natural polysaccharides including cashew gum, pectin, chitin, alginate, cellulose, starch, and chitosan (Ch) have gained attention due to their renewability, abundance, biocompatibility, biodegradability, environment-friendliness, and inexpensiveness [11,12]. Ch, which is an amino polysaccharide of high molecular weight and crystalline structure, is produced via alkaline deacetylation of chitin that is derived from exoskeletons of krills, lobsters, shrimps, crabs and crawfish. It is composed of binary linear units of (1→4)-2-amino-2-deoxy-β-d-glucan and (1→4)-2-acetamido-2-deoxy-β-d-glucan [1,13,14]. There are numerous remarkable characteristics of Ch including low-cost, chelating capacity, availability, anti-microbial activity, biodegradability, low immunogenicity and being environmentally benign [15]. However, pure Ch is known for its low specific gravity and poor mechanical integrity [16]. Graft copolymerization of vinyl monomers, which contain hydrophilic side groups, onto the Ch backbone has been known to enhance the biodegradability, pH resistance, thermal stability, water absorptive capability, and salt tolerance as well as minimize the need for oil-based monomers [1,17,18]. Acrylic acid (AA) as vinyl monomer has been extensively studied due to its decreased cytotoxicity and improved biocompatibility [19]. However, composite materials derived from natural polymers grafted with vinyl monomers suffer due to their high manufacturing cost and reduced mechanical strength [20]. 

Recently, incorporation of inorganic fillers such as clay minerals has yielded organic/inorganic composites with improved stiffness and strength, lower cost of production, better handling, enhanced swelling ability, and satisfactory adsorption performance [13,20,21,22,23]. Montmorillonite (Mt), which is a member of the smectite group, is comprised of a 2:1 ratio of silica tetrahedron and alumina octahedral sheets [24]. The formula of Mt is (Na,Ca)_0.33_(Al, Mg)_2_(Si_4_O_10_)(OH)_2_·*n*H_2_O, where it is described as a soft layered silicate with high cation exchange capacity, small particle diameter, and excellent aspect ratio and plane strength [25,26]. 

In the previous work of Abdel Aziz and Salama [27], Ch-graft-polyacrylic acid (ChPA) with Mt as filler material was prepared as a superabsorbent. The results showed the disappearance of d_001_ peak at 2θ = 6.6° after polymerization. This implies exfoliation of vinyl-modified Mt into individual nanolayers has occurred [27,28]. The maximum equilibrium swelling was determined via optimization study using response surface methodology (RSM) with Box-Behnken Design (BBD). The following optimal conditions were determined: 2.98 wt %. initiator, 1.04 wt % crosslinker and 9.76 AA/Ch molar ratio [27]. RSM is comprised of statistical and numerical methods utilized in the optimization of multiple factor levels in material preparation and treatment technologies [29,30]. The relationship between process variables and their responses are evaluated where an empirical polynomial model is fitted [31]. RSM also help recognize which of the main variables and their interactions significantly affect the process. However, there are no studies reported on the fuzzy logic technique in the field of material synthesis for superabsorbents. In literature, there is no information that utilized the fuzzy logic system to investigate the cumulative uncertainty error and variable cost on the preparation of superabsorbent. 

The fuzzy logic method is a precise modeling tool employed in the evaluation, mapping, modeling, and prediction in areas of environment and ecology [32]. Fuzzy logic allows adaptability of models with a degree of uncertainty near the boundary of unsuitable and suitable areas. The fuzzy system applies sigmoidal adjustment (Gaussian, linear or other method) to adequately define the system’s behavior for criteria parameterization [33]. Fuzzy logic system is less likely to be influenced by the analyst’s opinion where an acceptable model is defined by the minimum and maximum values of the variables [34].

The present work uses multi-objective optimization with the fuzzy logic concept in the selection of the optimum condition for the synthesis of Ch-Mt composites grafted with PA (ChPA-Mt). The global optimum solution that would yield the best swelling capacity of ChPA-Mt and provide the lowest possible variable cost was determined. The effect of AA/Ch molar ratio, ammonium persulphate as initiator and *N*,*N*’-methylenebisacrylamide as crosslinker on the total variable cost and swelling capacity with respect to the synthesis of ChPA-Mt was investigated. 

Using the fuzzy optimization approach, the selection of the ideal swelling capacity was dependent on the end-use (absorbent/adsorbent) of the material. Two cases were considered in the present work where the first case determined the appropriate condition to synthesize an absorbent that would yield the maximum swelling capacity for the least variable cost. The second case evaluated the synthesis of an adsorbent where minimal swelling capacity is preferred at the least possible cost. Superabsorbents have been utilized as an adsorbent in the removal of crystal violet dye [35], napthol green dye [35], sunset yellow dye [35], anionic dye [36], Cu^2+^ [37], Co^2+^ [37], Ni^2+^ [37], and Cd^2+^ [37] from wastewater. Adsorption technology involving industrial fixed-bed reactors requires adsorbent material to have the least swelling capacity to avoid fouling and clogging of the reactor [38].

## 2. Methodology

### 2.1. Materials

Acetic acid, Ch (90% deacetylation degree), Mt, *N*,*N*’-methylenebisacrylamide, ammonium persulphate, and AA were acquired from Sigma-Aldrich (WI, USA). The preparative method utilized for *N*-octyl-*N*-vinyl-2-pyrrolidonium bromide was based on previous work by Naguib et al. [39].

### 2.2. Experimental Method

Approximately 50 mL of acetic acid (1% v/v) was used to dissolve 2 g Ch, and the solution was purged for 40 min using N_2_ gas. The solution was added to a pre-determined amount of ammonium persulphate, after which AA and *N*,*N*’-methylenebisacrylamide were also added to the mixture. Then, mixture was allowed to react for 4 h at 70 °C. The final solution was allowed to cool, and was then neutralized with 1 M NaOH until a pH of 7.0 was attained. The ChPA-Mt material was washed with water to remove any impurities and dried in an oven at 70 °C. Unreacted AA and PA were removed using ethanol in a Soxhlet extractor for 24 h, ensuring that only crosslinked copolymer was attained.

### 2.3. Fuzzy Multi-Objective Optimization Method

**Figure 1:**
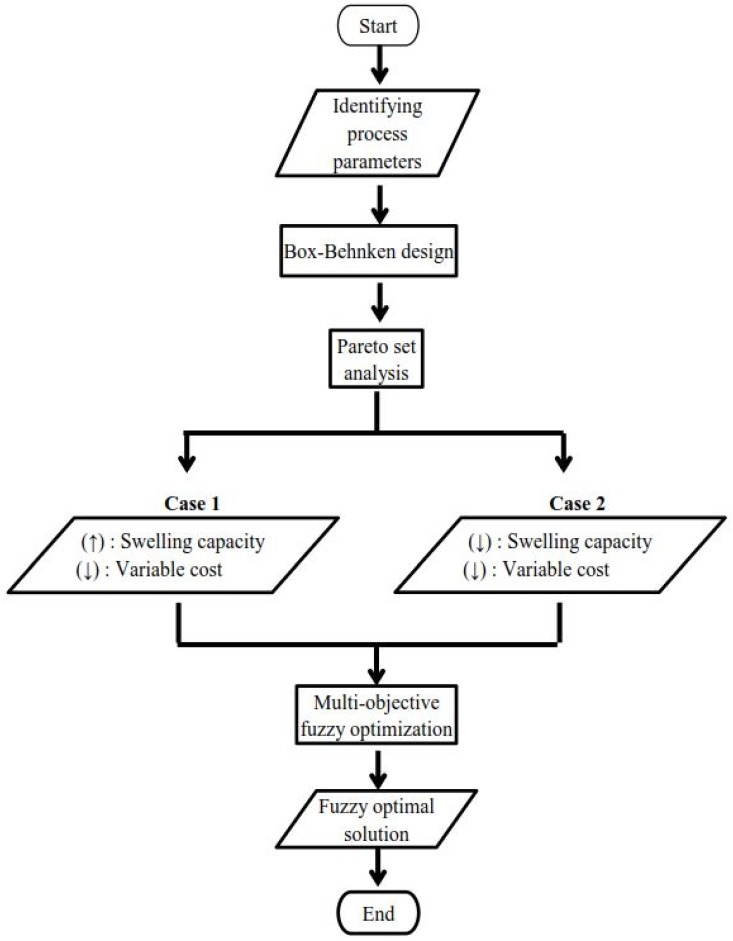
Fuzzy optimization process diagram.

The most probable parametric conditions of the variable cost and the total swelling capacity in account of its cumulative uncertainty of results was attained by applying the Pareto set analysis and fuzzy mathematical programming in the optimization studies. For the process parameters (vinylic monomer/biopolymer molar ratio, crosslinker and initiator) of the synthesis of a ChPA-Mt material, the process parameters and the material costs are listed in Table 1. Moreover, Table 2 shows the data derived from the study of Abdel Aziz and Salama [27] with the additional incorporation of the cumulative uncertainty of results. A linguistic value that applies the true value theory, ranging from completely false (0) to completely true (1), was utilized to estimate the reasoning approach in the fuzzy optimization analysis [40]. Figure 1 illustrates the algorithm flowchart of the fuzzy system in the present work. Optimization studies were performed using Lingo 18.0 (Lindo Systems, Chicago, IL, USA) where non-linear programming software uses a global optimizer.

#### 2.3.1. Determination of Boundary Limits through the Pareto Optimality 

The process of choosing an exact number of courses that would generate a substantial overall effect for numerous competing practical solutions was carried out using the Pareto analysis [41]. The initial Pareto optimum solution under several weights of the objective function was obtained using the Pareto set process [42]. Initially, the boundary limits for variable cost and swelling capacity were attained using the method. The multi-objective fuzzy optimization applied the boundary limits. The first objective function in Equation (1) pertains to the swelling capacity in consideration of its uncertainty error (*Y_Q_*: g/g). This is either to maximize or minimize depending on desired condition of the case study in the synthesis of the polymer clay nanocomposite material. For *Y_Q_* at its maximum value, the upper limits of *Y_Q_* and total variable cost (*VC_T_*: USD/kg) were determined. The lower boundary limits of *Y_Q_* and *VC_T_* were determined through minimizing the second objective function in Equation (2). In the case of determining the minimum value of *Y_Q_*, this was able to draw out the lower and upper limits of *Y_Q_* and *VC_T_*, respectively, while its upper and lower boundaries were determined through Equation (2).

Objective functions:

(1) $$\max\;/\min Y_{Q}$$

(2) $$\min VC_{T}$$

In order to satisfy the objective functions, the constraints were subjected to Equations (3)–(7). The sum of the swelling capacity (*Y*: g/g) and its corresponding uncertainty error (*W_Y_*: g/g) are shown in Equation (3). The response for the model equation in Equation (4) pertains to the swelling capacity of the synthesized polymer clay nanocomposite material. The incorporation of the constraints of the cumulative uncertainty of the response for the swelling capacity and the total variable cost are shown in Equations (5) and (6), respectively. Equation (7) pertains to the feasible regions of the variables tested based on the vinylic monomer/biopolymer molar ratio, crosslinker and initiator.

Constraints:

(3) $$Y_{Q}=Y+W_{Y}$$

(4) $$Y=\beta_{0}+\overset{n}{\underset{i=1}{\sum }}\beta_{i}X_{i}+\overset{n-1}{\underset{i=1}{\sum }}\overset{n}{\underset{i=1}{\sum }}\beta_{ij}X_{i}X_{j}+\overset{n}{\underset{i=1}{\sum }}\beta_{ii}X_{i}^{2}$$

(5) $$W_{Y}={\left(\overset{n}{\underset{i=n}{\sum }}\frac{\partial Y}{\partial X_{i}}W_{X_{i}}\right)}^{\frac{1}{2}}\;\;$$

(6) $$VC_{T}=\overset{n}{\underset{i=1}{\sum }}VC_{i}$$

(7) $$X_{i}^{L}\leq X_{i}\leq X_{i}^{U}$$

where $\beta_{0}$ pertains to the constant; *i* and *j* are the single and interacting factor indices, respectively; *n* denotes the tested number of variables; $\beta_{i}$, $\beta_{ij}$. and $\beta_{ii}$ are the regression coefficients; *X_i_* and *X_j_* are the independent variables; $W_{X}$. and *VC* are the variable uncertainty and variable cost, respectively; while *L* and *U* represent the lower and upper boundary limit, respectively.

#### 2.3.2. Multi-Objective Decision Making through Fuzzy Logic

The identification of the best solution derived from the recognized set of Pareto optimal conditions would require the utilization of a multi-criteria analysis [42]. The fuzzy mathematical programming system was employed to arrive at a solution from a multi-objective decision-making problem [43,44,45]. The perception of max–min aggregation that would concurrently enhance the satisfaction degree would be employed to maximize the overall satisfaction degree for the objective function [43,44,45,46,47,48]. Moreover, the degree of satisfaction for overall swelling capacity and the total variable cost of the results must satisfy the overall satisfaction as presented in Equation (8).

Objective function:

(8) $$\max\mu_{O}\leq \mu_{SC}\;and\;\mu_{VC}$$

where $\mu_{SC}$ and $\mu_{VC}$ are the individual level of satisfaction of the overall swelling capacity and the total variable cost, respectively.

The constraints used to attain the fuzzy goal are given in Equations (9)–(12). The linear membership function depending on the desired function in either maximizing or minimizing the swelling capacity for the polymer clay nanocomposite material are shown in Equations (9) and (10), respectively. Equation (11) refers to the linear membership function for minimizing the total variable cost involved in the material synthesis. Moreover, the level of satisfaction has a limiting constraint that could only be adjusted within a feasible range as described in Equation (12). 

Constraints:

(9) $$\mu_{SC}=\frac{Y_{Q}-Y_{Q}^{L}}{Y_{Q}^{U}-Y_{Q}^{L}}$$

(10) $$\mu_{SC}=\frac{Y_{Q}^{U}-Y_{Q}}{Y_{Q}^{U}-Y_{Q}^{L}}$$

(11) $$\mu_{VC}=\frac{VC_{T}{}^{U}-VC_{T}}{VC_{T}{}^{U}-VC_{T}{}^{L}}$$

(12) $$\mu_{O}^{L}\leq \mu_{O}\leq \mu_{O}^{U}$$

## 3. Results and Discussion

### 3.1. Effect of AA/Ch Molar Ratio on the Swelling Capacity and Its Variable Cost

**Figure 2:**
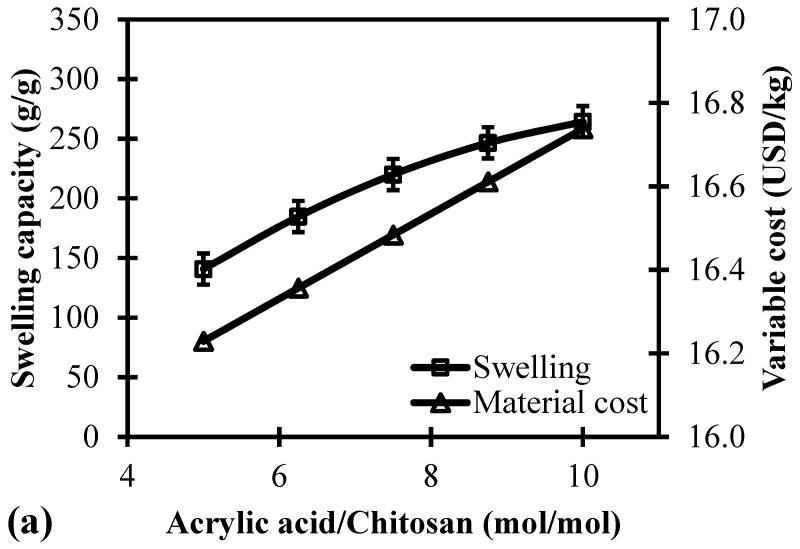

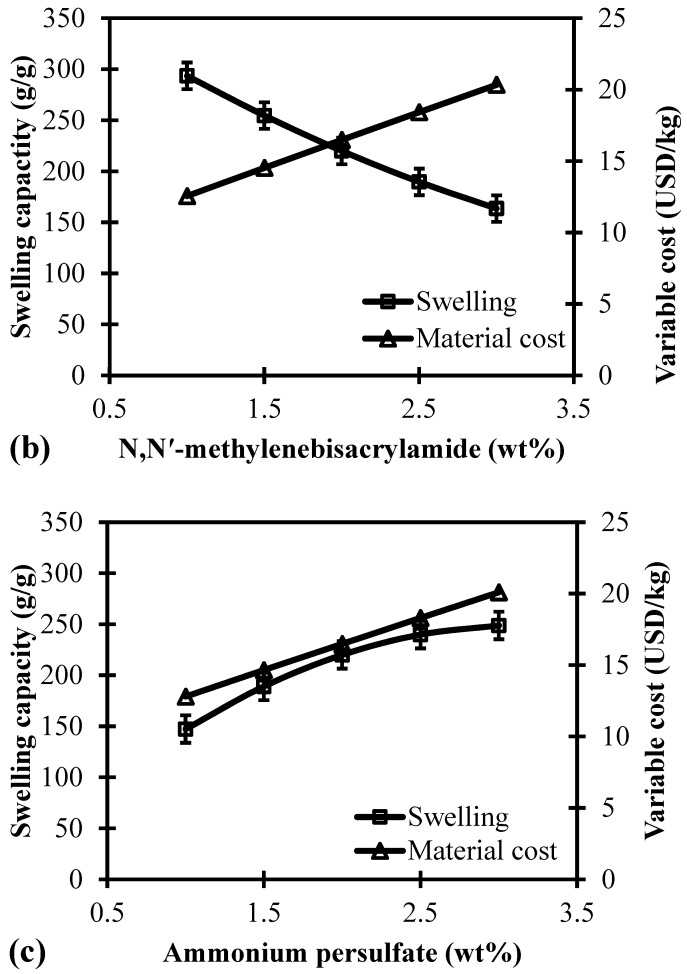
Simultaneous analysis of swelling capacity and variable cost at different levels of (**a**) acrylic acid/chitosan, (**b**) *N*,*N*′-methylenebisacrylamide, and (**c**) ammonium persulfate.

In order to investigate the influence of AA/Ch molar ratio on the material synthesis, the amount of *N*,*N*′-methylenebisacrylamide and ammonium persulfate were individually kept constant at 2 wt%. The identification of a proper ratio between the vinylic monomer and biopolymer is essential to avoid the competition with AA led to its auto-polymerization aside from the grafting of the Ch material [49]. Figure 2a illustrates the effect of AA/Ch molar ratio towards the swelling capacity and variable cost.

The trend of the molar ratio from 5 to 10 resulted in a swelling capacity from 141 ± 15 g/g to 264 ± 11 g/g, respectively. It was observed that a higher AA concentration leads to a larger swelling capacity upon the synthesis of the polymer clay nanocomposite material. An electrostatic repulsion and ionization in the chain takes place at a higher ratio due to the promotion of available carboxylic groups that is able to expand the originally coiled molecules [50].

In the aspect of the incurred total variable cost, the material synthesis at incremental molar ratio from 5 to 10 showed higher variable cost from 16.2 USD/kg to 16.7 USD/kg. This is due to a higher AA loading that is utilized to synthesize the polymer clay nanocomposite material. Aside from achieving the desirable swelling capacity, it is also essentially important to minimize the variable cost to achieve an efficient and cheap material according to its function. 

### 3.2. Effect of N,N′-methylenebisacrylamide on the Swelling Capacity and Its Variable Cost

*N*,*N*’-methylenebisacrylamide is used as the crosslinker for the polymer clay nanocomposite material synthesis. Crosslinking is favorable due to its capacity to improve chemical stability and mechanical strength [51]. To determine the effect of the amount of *N*,*N*’-methylenebisacrylamide on the resulting material, the AA/Ch molar ratio of 7.5 and ammonium persulfate content of 2 wt% were kept constant throughout the variation of the crosslinker dosage. The detailed response of the swelling capacity and variable cost at different *N*,*N*’-methylenebisacrylamide concentration are depicted in Figure 2b.

At 1 wt % *N*,*N*’-methylenebisacrylamide, a swelling capacity of 294 ± 15 g/g was synthesized. Increasing the crosslinker content further to 3 wt % was able to lower the swelling capacity to 164 ± 12 g/g. A higher crosslinker concentration was attributed to the decreasing polymer chain relaxation that led to less swelling [50]. Furthermore, large quantities of crosslinker content increased the crosslink density that tended to restrict additional free volume from being utilized [52].

For the trend from 1 wt % to 3 wt % of *N*,*N*’-methylenebisacrylamide, the swelling capacity decreased while variable cost increased. The inverse relationship of the resulting material and its cost proved to be one of the essential criteria for achieving an efficient outcome for its synthesis. Higher *N*,*N*’-methylenebisacrylamide implies more material usage that increases the specified variable cost the specified parameter. After selecting the appropriate combination of the crosslinker dosage, the resulting polymer clay nanocomposite material could be utilized in various applications due to its ability to form macromolecular structures [51].

### 3.3. Effect of Ammonium Persulfate on the Swelling Capacity and Its Variable Cost

The use of chemical initiators is essential for its function in generating living free radicals that enable the initiation of polymerization reactions in vinyl monomers [53]. Ammonium persulfate is used as the initiator in the graft polymerization of the AA onto Ch. To determine the effects of ammonium persulfate, the AA/Ch molar ratio and *N*,*N*’-methylenebisacrylamide dosage were kept constant at 7.5 and 2 wt %, respectively. Figure 2c shows the results of the variation of ammonium persulfate on the swelling capacity and variable cost.

The results exhibited increasing swelling capacity from 147 g/g to 249 g/g at the corresponding ammonium persulfate dosages from 1 wt % to 3 wt %. In terms of the pore distribution, a higher initiator content provides additional free volume that allows higher water mobility leading to a higher swelling degree [52]. Furthermore, an increase in the initiator concentration is able to promote the formation of polymer chain ends in the network leading to higher swelling activities [54]. 

Aside from the crosslinker, the use of the initiator also contributes to a high production cost in the synthesis of the polymer clay nanocomposite material [49]. The variable cost increased from 12.8 USD/kg to 20.1 USD/kg when ammonium persulfate was changed from 1 wt % to 3 wt %. Higher initiator content increased the material loading that mainly contributed to a larger variable cost. Thus, this is an essential factor for consideration to achieve optimal results upon determining the best compromise solution in a low-cost material synthesis at its ideal swelling capacity.

### 3.4. Pareto Set Result

The Pareto set analysis determined the appropriate upper and lower boundary limits of the swelling capacity with its cumulative uncertainty error and the total variable cost of the polymer clay nanocomposite material. The BBD under the response surface methodology was utilized as the basis to draw out the Pareto efficiency upon the grafting of AA onto Ch. The results of the analysis would then be essentially used towards the preference criterion in the latter part of the decision-making analysis. In the material synthesis of the two case studies, a non-linear model equation is used for the response of the swelling capacity. This type of equation often leads to multiple local optimums and the global optimum are not directly given [55]. The global optimal solution is favorable to carry out the best possible solution in a given set of constraints for the objective function. Thus, the global optimizer option in the Lingo software is utilized to be able to achieve the best resulting solution for synthesis.

The Pareto analysis generates a Ch-graft-PA material through the application of Equations (13)–(27). Equation (13) shows the generated and validated model equation adapted from the study of Abdel Aziz and Salama [27] that describes the swelling capacity of the produced polymer clay nanocomposite material. The cumulative uncertainty of the response in Equation (14) indicates the possible error associated with the synthesis step towards the desired value of the swelling capacity. This is specifically determined through the partial derivatives with respect to AA/Ch molar ratio (Equation (15)), *N*,*N*’-methylenebisacrylamide concentration (Equation (16)), and ammonium persulfate concentration (Equation (17)). The integration of the incurred total variable cost in Equation (18) is a key parameter in the Pareto optimal analysis. Specifically, the individual variable cost of AA, *N*,*N*’-methylenebisacrylamide and ammonium persulfate are shown in Equations (19)–(21), while its calculated material usage are presented in Equations (22)–(24), respectively. Furthermore, Equations (25)–(27) are the boundary limits that denote the respective variables of AA, *N*,*N*’-methylenebisacrylamide and ammonium persulfate.

(13) $$Y=-7.25+47.9X_{1}-153X_{2}+122.25X_{3}+7.2X_{1}X_{2}+2.2X_{1}X_{3}-2.8X_{1}X_{1}+8.5X_{2}X_{2}-22X_{3}X_{3}$$

(14) $$W_{Y}=\sqrt{{\left(\frac{\partial Y_{Q}}{\partial X_{1}}W_{X_{1}}\right)}^{2}+{\left(\frac{\partial Y_{Q}}{\partial X_{2}}W_{X_{2}}\right)}^{2}+{\left(\frac{\partial Y_{Q}}{\partial X_{3}}W_{X_{3}}\right)}^{2}}$$

(15) $$\frac{\partial Y}{\partial X_{1}}=47.9+7.2X_{2}+2.2X_{3}-5.6X_{1}$$

(16) $$\frac{\partial Y}{\partial X_{2}}=-153+7.2X_{1}+17X_{2}$$

(17) $$\frac{\partial Y}{\partial X_{3}}=122.25+2.2X_{1}-44X_{3}$$

(18) $$VC_{T}=VC_{1}+VC_{2}+VC_{3}$$

(19) $$VC_{1}=\frac{442.42A}{\left(A+I+C+2\right)}$$

(20) $$VC_{2}=\frac{421.43I}{\left(A+I+C+2\right)}$$

(21) $$VC_{3}=\frac{395.96C}{\left(A+I+C+2\right)}$$

(22) $$A=4.804x10^{-4}X_{1}$$

(23) $$I=\frac{\left(A+2\right)X_{2}}{100}$$

(24) $$C=\frac{\left(A+2\right)X_{3}}{100}$$

(25) $$5\leq X_{1}\leq 10$$

(26) $$1\leq X_{2}\leq 3$$

(27) $$1\leq X_{3}\leq 3$$

where *X_1_*, *X_2_* and *X_3_* are the variables of AA/Ch molar ratio; *N*,*N*’-methylenebisacrylamide concentration (wt %) and ammonium persulfate concentration (wt %), respectively; $W_{X_{1}}$, $W_{X_{2}}$, and $W_{X_{3}}$ are the uncertainty of the tested variables; *VC_T_*, *VC_1_*, *VC_2_*, and *VC_3_* are the total variable cost, and the individual variable cost of AA, *N*,*N*’-methylenebisacrylamide, and ammonium persulfate, respectively, in USD/kg; *A*, *I* and *C* are the computed material usage for AA, *N*,*N*’-methylenebisacrylamide, and ammonium persulfate in g.

#### 3.4.1. Case 1: Absorbent Material

To determine the upper boundary limits of the swelling capacity and total variable cost of the Ch-graft-PA absorbent material, the set objective function for the summation of Equations (13) and (14) were maximized. This is subject to the constraints in Equations (15)–(27) that must be satisfied. Results indicated a swelling capacity at a global maximum of 354 g/g and a 10 g/g cumulative error of uncertainty. This is under the parametric conditions of 10 AA/Ch molar ratio, 1.0 wt % *N*,*N*’-methylenebisacrylamide, and 3.0 wt % ammonium persulfate with an associated total variable cost of 16.5 USD/kg.

A separate objective function was set to determine the lower boundary limits of the swelling capacity and total variable cost. Equation (18) is minimized under the established conditions of Equations (13)–(17) and (19)–(27). The results of the minimum swelling capacity (165 g/g) and total variable cost (8.5 USD/kg) were obtained in the parameters of 5 AA/Ch molar ratio, 1.0 wt % *N*,*N*’-methylenebisacrylamide, and 1.0 wt % ammonium persulfate. Furthermore, its cumulative error of uncertainty reached 21 g/g.

**Figure 3:**
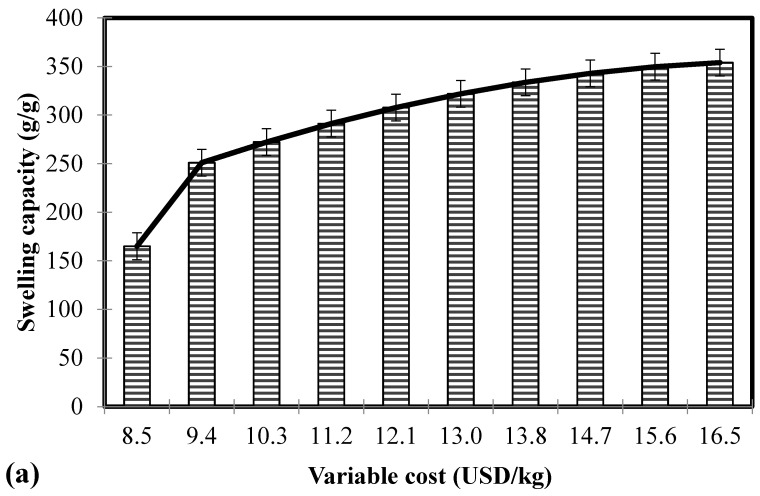

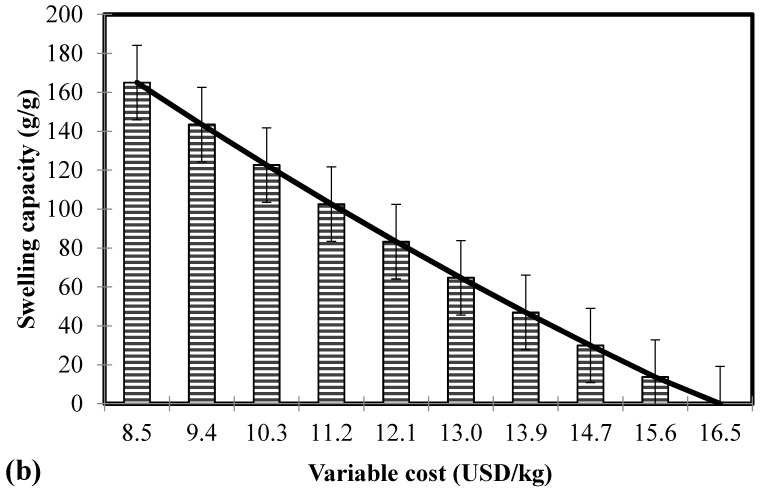
Pareto plot of variable cost with (**a**) maximum and (**b**) minimum conditions on the swelling capacity.

Figure 3a depicts the graphical representation of the Pareto optimality analysis that identified the appropriate boundary limits through determining the maximum swelling capacity at the least possible cost for the material synthesis of Ch-graft-PA absorbent. Results from the Pareto analysis revealed that large swelling capacities incurred higher variable cost. The swelling capacity is characterized by the amount of absorbed water and absorption rate [56]. Synthesizing absorbents with large swelling capacities are favorable to function as a superabsorbent polymer. Typically, the preparation of an AA based superabsorbent utilizes the initiator and crosslinker that contributes to higher production cost [49]. Higher AA/Ch molar ratio and ammonium persulfate concentration facilitate more carboxylic groups that initiate additional sites for crosslinking reaction, while lower *N*,*N*’-methylenebisacrylamide concentration would avoid an extensive crosslinking to favorably increase the swelling characteristic [27]. Thus, a proper compromise between a high swelling capacity and a low variable cost is noteworthy for further investigation. 

#### 3.4.2. Case 2: Adsorbent Material

The sum of Equations (13) and (14) are minimized subject to the constraints in Equations (15)–(27) to appropriately establish the lower bound of the swelling capacity according to the desired functionality of the adsorbent material. Results for the global minimum value showed a non-swelling material that has a total variable cost of 16.5 USD/kg (upper bound for the total variable cost). The parametric conditions ensued five AA/Ch molar ratio, 3.0 wt % *N*,*N*’-methylenebisacrylamide and 1.0 wt % ammonium persulfate. Furthermore, the cumulative error of uncertainty in the swelling capacity reached 18 g/g.

On the other hand, the objective function in Equation (18) is minimized under the conditions of Equations (13)–(17) and (19)–(27). This resulted to an upper boundary limit of the swelling capacity at 165 g/g (with a 21 g/g cumulative uncertainty of results) and a lower boundary limit of the total variable cost at 8.5 USD/kg for the synthesis of Ch-graft-PA adsorbent. The variables of acid/Ch molar ratio, *N*,*N*’-methylenebisacrylamide concentration and ammonium persulfate concentration rendered values of 5.0, 1.0 wt %, and 1.0 wt %, respectively.

The results of the Pareto set that comprise the boundary limits to appropriately synthesize the adsorbent material according to its desired minimum swelling capacity and variable cost are depicted in Figure 3b. In adsorption processes, high swelling materials have the capacity to adsorb large quantities of water molecules that increases the volume of the adsorbent. This would consequently lead to fouling and a sudden stop in the flow of wastewater in the column after a fixed amount of time [38]. It is thus desirable for adsorbents to have a low swelling capacity to be able to capitalize on the material in industrial applications. However, it was revealed in the results that a lower swelling characteristic incurs a higher total variable cost in the Pareto analysis. This is attributed to a higher material usage of the *N*,*N*’-methylenebisacrylamide to lower the swelling capacity due to extensive crosslinking in the adsorbent synthesis [27]. Therefore, an investigation in a multi-objective optimization using the fuzzy logic approach is essential for a decision making analysis.

### 3.5. Multi-Objective Fuzzy Optimization Result

In order to optimize the objective function and the constraints, a decision in a fuzzy environment is required through the analogy of nonfuzzy environments as to select the activities that enables to simultaneously satisfy the objective function and constraints [57]. Based on the functionality of the polymer clay nanocomposite material, the fuzzy optimization was utilized for the decision making strategy of the two case studies presented in this research. The main objective function in Equation (28) is carried out in a global optimization procedure under the interval of 0 (dissatisfied) to 1 (satisfied) of the overall satisfaction rating ($\mu_{O}$) in Equation (29). The multi-objective fuzzy optimization process also included the constraints in Equations (13)–(27).

(28) $$\mu_{O}\leq \mu_{SC}\;\&\;\mu_{VC}$$

(29) $$0\leq \mu_{O}\leq 1$$

In order to reach the fuzzy goal of the synthesis of an absorbent material (Case 1), the fuzzy constraints are subject to the constraints on the maximum swelling capacity ($\mu_{SC}$) and minimum variable cost ($\mu_{VC}$) in Equations (30) and (31), respectively.

(30) $$\mu_{SC}=\frac{Y_{Q}-185.58}{178.23}$$

(31) $$\mu_{VC}=\frac{16.47-VC_{T}}{7.96}$$

For the adsorbent synthesis (Case 2), the fuzzy goal is attained with the minimized fuzzy constraints of the overall swelling capacity (Equation (32)) and total variable cost (Equation (33)).

(32) $$\mu_{SC}=\frac{185.58-Y_{Q}}{168.69}$$

(33) $$\mu_{VC}=\frac{16.49-VC_{T}}{7.94}$$

**Figure 4:**
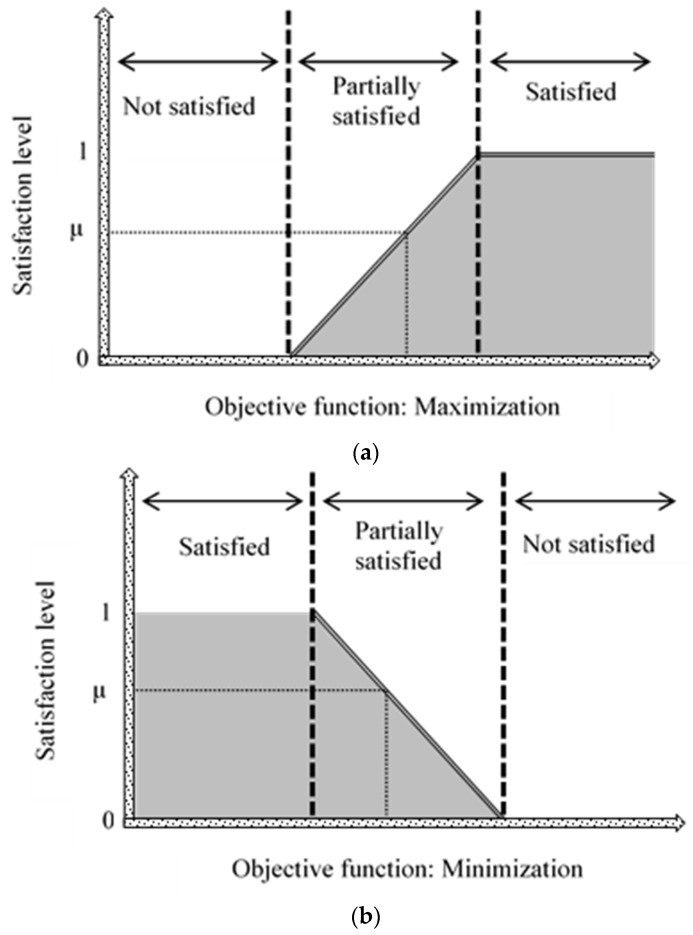
Linear membership function at goals towards (**a**) maximization and (**b**) minimization.

The fuzzy objective function and its constraints are characterized by its membership function. Specifically, the linear membership function can facilitate the estimation of non-linear equations and is favorable to define the subjective preference of any objective uncertainty [58]. In a graphical representation, the linear membership function can be either maximized (Figure 4a) or minimized (Figure 4b) according to its objective function. Moreover, the boundary limits utilized in the fuzzy optimization for the swelling capacity and variable cost are derived from the Pareto analysis described in the preceding section. The boundary limits for absorbent synthesis (Case 1) are as follows: (1) overall swelling capacity of 364 g/g (upper), (2) overall swelling capacity of 186 g/g (lower), (3) total variable cost of 16.5 USD/kg (upper), and (4) total variable cost of 8.5 USD/kg (lower). For adsorbent synthesis (Case 2), the boundary limits are as follows: (1) overall swelling capacity of 186 g/g (upper), (2) overall swelling capacity of 17 g/g (lower), (3) total variable cost of 16.5 USD/kg (upper), and (4) total variable cost of 8.5 USD/kg (lower).

The fuzzy global optimal results from the clay–polymer composite material synthesis are listed in Table 3. Results showed that the maximum *λ_O_* values for the absorbent and adsorbent material were 0.672 and 0.523, respectively. This indicates that the material synthesis upon the various matrix of AA/Ch molar ratio, *N*,*N*′-methylenebisacrylamide concentration, and ammonium persulfate concentration were able to successfully reach a compromise solution for the absorbent and adsorbent synthesis with an aggregated satisfaction rating of 67.2 % and 52.3 %, respectively. 

For the absorbent synthesis (Case 1), a proper allocation scheme that achieves the fuzzy goal subjected to its fuzzy constraints reached an overall swelling capacity of 305 g/g and a total variable cost of 10.8 USD/kg. The solution that achieved the highest swelling capacity at the least possible cost corresponds to the fuzzy optimum conditions of 10.0 AA/Ch molar ratio, 1.0 wt % *N*,*N*′-methylenebisacrylamide, and 1.6 wt % ammonium persulfate. The resulting solution is desirable for synthesizing a superabsorbent material with a high swelling capacity. Typically, superabsorbents are able to keep large quantity of water that are irremovable under pressure due to its loosely crosslinked hydrophilic characteristic as a polymer material. A high-level acid to polymer ratio, low-level crosslinker dose, and mid-level initiator dose are desirable to appropriately initiate a loose crosslinking reaction in the carboxylate groups. In comparison with the study of Abdel Aziz and Salama [27], the optimum results using the RSM model led to a swelling capacity of 351 g/g (9.76 AA/Ch molar ratio, 1.04 wt % N′-methylenebisacrylamide, and 2.98 wt % ammonium persulfate). However, the total variable cost for this synthesis is 16.6 USD/kg that is 53.7% more expensive than in the fuzzy optimal solution. Thus, fuzzy optimization was able to reach a compromise solution based on a desirable swelling capacity with a more economical cost for superabsorbent synthesis.

Conversely, materials in adsorption technology that exhibit a high swelling degree are highly unfavorable as adsorbents [38]. The adsorbent synthesis (Case 2) in the fuzzy-based decision making analysis revealed a minimum overall swelling capacity and total variable cost of 97 g/g and 12.3 USD/kg, respectively. The fuzzy optimum solution resulted in the parameters of 5.0 AA/Ch molar ratio, 1.9 wt % *N*,*N*′-methylenebisacrylamide, and 1.0 wt % ammonium persulfate. This has been selected to reach a compromise solution by simultaneously minimizing the swelling capacity and its associated costs for synthesis. The acid to polymer ratio (low), crosslinker (mid), and initiator (low) combination are favorable due to promoting an extensive crosslinking reaction leading to smaller swelling capacity in the Ch-graft-PA material. This would ultimately satisfy the functionality of the adsorbent as the swelling characteristic is a crucial factor in adsorption technologies.

## 4. Conclusions

In this study, a strategic decision making analysis for synthesis of ChPA-Mt was investigated depending on its functionality as an absorbent or adsorbent. The tested variables of AA/Ch molar ratio, *N*,*N*′-methylenebisacrylamide concentration, and ammonium persulfate concentration were studied in conjunction with an uncertainty analysis in the response (swelling capacity) and its associated variable cost. The Pareto set analysis indicated the lower to upper boundary limits for the two case studies as follows: (1) absorbent material, overall swelling capacity (186 g/g to 364 g/g) and total variable cost (8.5 USD/kg to 16.5 USD/kg); and (2) adsorbent material, overall swelling capacity (17 g/g to 186 g/g) and total variable cost (8.5 USD/kg to 16.5 USD/kg). A multi-objective fuzzy optimization showed an innovative approach to determine a solution for the best condition in the material synthesis. According to its functionality, the absorbent material (305 g/g and 10.8 USD/kg) and adsorbent material (97 g/g and 12.3 USD/kg) showed a satisfaction rating in its synthesis of 67.2% and 52.3%, respectively. Therefore, the application of the global fuzzy optimal solutions and its respective conditions in the case studies proves to be efficient in relation to the material synthesis. Furthermore, the incorporation of the criteria of the variable cost in terms of material usage and the cumulative uncertainty of the response have successfully ensued essential compromise results in the decision making process.

## Figures and Tables

**Table 1:** (**a**) Variables and (**b**) costing parameters for the synthesis of the clay-polymer composite material.

**(a) Process Parameters**	**Unit**	**Range**
AA/Ch (*A*)		5–10
*N*,*N*′-methylenebisacrylamide (*B*)	wt %	1–3
Ammonium persulfate (*C*)	wt %	1–3
**(b) Chemicals**	**Unit**	* Material Cost
AA	USD/g	0.44
*N*,*N*′-methylenebisacrylamide	USD/g	0.41
Ammonium persulfate	USD/g	0.39

* Sigma Aldrich (WI, USA); AA: acrylic acid; Ch: chitosan.

**Table 2:** Clay–polymer composite material synthesis based on the BBD matrix.

Run	AA/Ch (*A*)	*N*,*N*′-methylenebisacrylamide (*B*: wt %)	Ammonium Persulfate (*C*: wt %)	Predicted Swelling Capacity (g/g)	* Cumulative Uncertainty (g/g)
1	5	2	1	74	19
2	5	3	2	66	14
3	5	1	2	232	17
4	5	2	3	164	14
5	7.5	3	3	192	9
6	7.5	2	2	220	13
7	7.5	2	2	220	13
8	7.5	3	1	91	17
9	7.5	2	2	220	13
10	7.5	1	3	322	13
11	7.5	1	1	221	19
12	7.5	2	2	220	13
13	7.5	2	2	220	13
14	10	3	2	226	10
15	10	2	1	186	17
16	10	1	2	320	13
17	10	2	3	299	8

Adapted from Abdel Aziz and Salama [27]; * calculated cumulative uncertainty of the results; BBD: Box-Behnken design.

**Table 3:** Fuzzy optimal solutions based on different objectives for the synthesis of ChPA-Mt material.

Parameters	Unit	Goal	
		Absorbent (↑): Swelling Capacity (↓): Variable Cost	Adsorben (↓): Swelling Capacity (↓): Variable Cost
$\mu_{O}$	%	67.2	52.3
$\mu_{SC}$	%	67.2	52.3
$\mu_{VC}$	%	67.2	52.3
Swelling capacity $\left(Y_{Q}\right)$	g/g	2	78
Cumulative uncertainty $\left(W_{Y_{Q}}\right)$	g/g	15	19
Overall $\left(Y_{Q}+W_{Y_{Q}}\right)$	g/g	305	97
Costing:			
Arylic acid	USD/kg	1.0	0.5
*N*,*N*′-methylenebisacrylamide	USD/kg	4.1	8.0
Ammonium persulfate	USD/kg	5.7	3.8
Total variable cost	USD/kg	10.8	12.3
Process Parameters:			
AA/Ch		10.0	5.0
*N*,*N*′-methylenebisacrylamide	wt %	1.0	1.9
Ammonium persulfate	wt %	1.6	1.0
